# The effects of continuous venovenous hemofiltration on coagulation activation

**DOI:** 10.1186/cc5080

**Published:** 2006-10-27

**Authors:** Catherine SC Bouman, Anne-Cornélie JM de Pont, Joost CM Meijers, Kamran Bakhtiari, Dorina Roem, Sacha Zeerleder, Gertjan Wolbink, Johanna C Korevaar, Marcel Levi, Evert de Jonge

**Affiliations:** 1 Department of Intensive Care, Academic Medical Center, University of Amsterdam, PO 22660, 1100 DD Amsterdam, The Netherlands; 2 Department of Vascular Medicine, Academic Medical Center, University of Amsterdam, The Netherlands; 3 Laboratory for Experimental and Clinical Immunology, Sanquin Blood Supply Foundation, Amsterdam, The Netherlands; 4 Department of Clinical Epidemiology & Biostatistics, Academic Medical Center, University of Amsterdam, The Netherlands; 5 Department of Internal Medicine, Academic Medical Center, University of Amsterdam, The Netherlands

## Abstract

**Introduction:**

The mechanism of coagulation activation during continuous venovenous hemofiltration (CVVH) has not yet been elucidated. Insight into the mechanism(s) of hemostatic activation within the extracorporeal circuit could result in a more rational approach to anticoagulation. The aim of the present study was to investigate whether CVVH using cellulose triacetate filters causes activation of the contact factor pathway or of the tissue factor pathway of coagulation. In contrast to previous studies, CVVH was performed without anticoagulation.

**Methods:**

Ten critically ill patients were studied prior to the start of CVVH and at 5, 15 and 30 minutes and 1, 2, 3 and 6 hours thereafter, for measurement of prothrombin fragment F1+2, soluble tissue factor, activated factor VII, tissue factor pathway inhibitor, kallikrein–C1-inhibitor and activated factor XII–C1-inhibitor complexes, tissue-type plasminogen activator, plasminogen activator inhibitor type I, plasmin–antiplasmin complexes, protein C and antithrombin.

**Results:**

During the study period the prothrombin fragment F1+2 levels increased significantly in four patients (defined as group A) and did not change in six patients (defined as group B). Group A also showed a rapid increase in transmembrane pressure, indicating clotting within the filter. At baseline, the activated partial thromboplastin time, the prothrombin time and the kallikrein–C1-inhibitor complex and activated factor XII–C1-inhibitor complex levels were significantly higher in group B, whereas the platelet count was significantly lower in group B. For the other studied markers the differences between group A and group B at baseline were not statistically significant. During CVVH the difference in the time course between group A and group B was not statistically significant for the markers of the tissue factor system (soluble tissue factor, activated factor VII and tissue factor pathway inhibitor), for the markers of the contact system (kallikrein–C1-inhibitor and activated factor XII–C1-inhibitor complexes) and for the markers of the fibrinolytic system (plasmin–antiplasmin complexes, tissue-type plasminogen activator and plasminogen activator inhibitor type I).

**Conclusion:**

Early thrombin generation was detected in a minority of intensive care patients receiving CVVH without anticoagulation. Systemic concentrations of markers of the tissue factor system and of the contact system did not change during CVVH. To elucidate the mechanism of clot formation during CVVH we suggest that future studies are needed that investigate the activation of coagulation directly at the site of the filter. Early coagulation during CVVH may be related to lower baseline levels of markers of contact activation.

## Introduction

Acute renal failure requiring renal replacement therapy occurs in approximately 4% of patients admitted to the intensive care unit, and often these patients are treated with some form of continuous renal replacement therapy (CRRT) [1]. CRRT requires anticoagulation to allow the passage of blood through the extracorporeal circuit over a prolonged period. Maintenance of CRRT circuits for sufficient duration is important for efficacy, cost-effectiveness and minimization of blood component loss. On the other hand, the systemic anticoagulation techniques used to prevent clotting of the circuit are important causes of morbidity in CRRT. Understanding the mechanisms involved in premature clotting of the filtration circuit is mandatory to optimize anticoagulation and to maintain filter patency.

Several studies have addressed the pathophysiology of circuit thrombogenesis, but the exact mechanism by which it occurs has not yet been elucidated. Multiple factors may play a role: the extracorporeal circuit itself, treatment modalities, platelet factors, coagulation factors, natural anticoagulants and fibrinolysis [2,3]. Clotting of CRRT circuits could be caused by increased activation of coagulation, initiated either by the (intrinsic) contact activation pathway or the (extrinsic) tissue factor/activated factor VII (FVIIa) pathway, or by low activity of the endogenous anticoagulant pathways, such as the antithrombin system, the protein C/protein S system and the tissue factor pathway inhibitor system. In addition, decreased fibrinolysis could also contribute to clotting of extracorporeal circuits.

Although much is known about the effect of a single hemodialysis treatment on the coagulation system, very few prospective studies have monitored the effects of repeated passage of blood through a CRRT circuit, and these studies were always performed with concurrent administration of anticoagulants, usually unfractionated heparin or low molecular weight heparin [4-7]. As heparin influences tissue-factor-mediated coagulation, contact-activated coagulation [7] and fibrinolysis [8], however, studies on the activation of coagulation during CRRT should ideally be performed without anticoagulation.

In the present study in critically ill patients with acute renal failure, we studied the effects of continuous venovenous hemofiltration (CVVH) without the use of anticoagulation on the activation of coagulation and fibrinolysis.

## Materials and methods

### Patients

The study was approved by the institutional review board and written informed consent was obtained from all participants or their authorized representatives. A cohort of 10 critically ill patients with acute renal failure requiring CVVH was studied. Patients were excluded if they fulfilled one of the following criteria: treatment with coumarins or platelet aggregation inhibitors within one week prior to starting CVVH; unfractionated heparin within 12 hours prior to starting CVVH or low molecular weight heparin within 48 hours prior to starting CVVH; treatment with extracorporeal techniques within 48 hours prior to starting CVVH; or discontinuation of CVVH for any reason other than clotting of the circuit (for example, transfer for a computed tomography scan).

### Continuous venovenous hemofiltration

Vascular access was obtained by insertion of a 14 F double-lumen catheter (Duo-Flow 400 XL; Medcomp, Harleysville, PA, USA) into a large vein (femoral, subclavian or internal jugular vein). Hemofiltration was performed with computer-controlled, fully automated hemofiltration machines (Diapact; Braun AG, Melsungen, Germany). A 1.9 m^2 ^cellulose triacetate hollow-fiber membrane with a sieving coefficient for β_2_-microglobulin of approximately 0.82 was used (CT190G; Baxter, McGaw Park, IL, USA). The blood flow rate was 150 ml/minute and warmed substitution fluid was added in predilution mode at a flow rate of 2 l/hour. The hemofiltration run continued until the extracorporeal circuit clotted. No anticoagulant was used during CVVH, and neither was the extracorporeal circuit primed with any anticoagulant.

### Blood collection

Blood was drawn from the venous limb of the hemofiltration catheter before starting hemofiltration, and at 5, 15 and 30 minutes and at 1, 2, 3 and 6 hours after commencement of CVVH. For the determination of contact activation 4.8 ml blood was collected in siliconized vacutainer tubes, to which 0.2 ml of a mixture of ethylenediamine tetraacetic acid (0.25 M), benzamidine (0.25 M) and soybean–trypsin inhibitor (0.25%) was added to prevent *in vitro *contact activation and clotting. All other blood samples were collected in citrated vacutainer tubes. Plasma was prepared by centrifugation of blood twice at 2500 × *g *for 20 minutes at 16°C, followed by storage at -80°C until assays were performed.

### Assays

The plasma concentrations of prothrombin fragment F1+2 (F1+2) were measured by ELISA (Dade Behring, Marburg, Germany). Soluble tissue factor was also determined by ELISA (American Diagnostica, Greenwich, CT, USA). The plasma concentration of FVIIa was determined on a Behring Coagulation System (Dade Behring) with the StaClot VIIa-rTF method from Diagnostica Stago (Asnières-sur-Seine, France). The tissue factor pathway inhibitor (TFPI) activity was measured on the Behring Coagulation System (Dade Behring) as described by Sandset and colleagues [9]. Kallikrein–C1-inhibitor and activated factor XII (FXIIa)–C1-inhibitor complexes were measured as described by Nuijens and colleagues [10]. Tissue-type plasminogen activator (t-PA) antigen and plasminogen activator inhibitor type I antigen were assayed by ELISA (Innotest PAI-1; Hyphen BioMed, Andrésy, France). Antithrombin activity was determined with Berichrom Antithrombin (Dade Behring) on a Behring Coagulation System (Dade Behring). Plasmin–antiplasmin (PAP) complexes were determined with a PAP micro ELISA kit (DRG, Berlin, Germany). Protein C was determined using the Coamatic protein C activity kit from Chromogenix (Mölndal, Sweden).

### Statistical analysis

Values are presented as the median (range). We used the Mann-Whitney U test to analyze the difference between baseline variables, and we used linear mixed models to evaluate the difference over time between groups. Data were analyzed using the Statistical Package for the Social Sciences for Windows (version 11.0; SPSS, Chicago IL, USA). *P *< 0.05 was considered significant. Hemofilter survival times were compared using the Kaplan–Meier method and the log-rank test (GraphPad Prism 4.0; GraphPad software Inc., San Diego CA, USA).

## Results

### Baseline characteristics

The baseline characteristics of the 10 enrolled patients are presented in Table 1.

### Thrombin generation and clotting of the circuit

**Figure 1:**
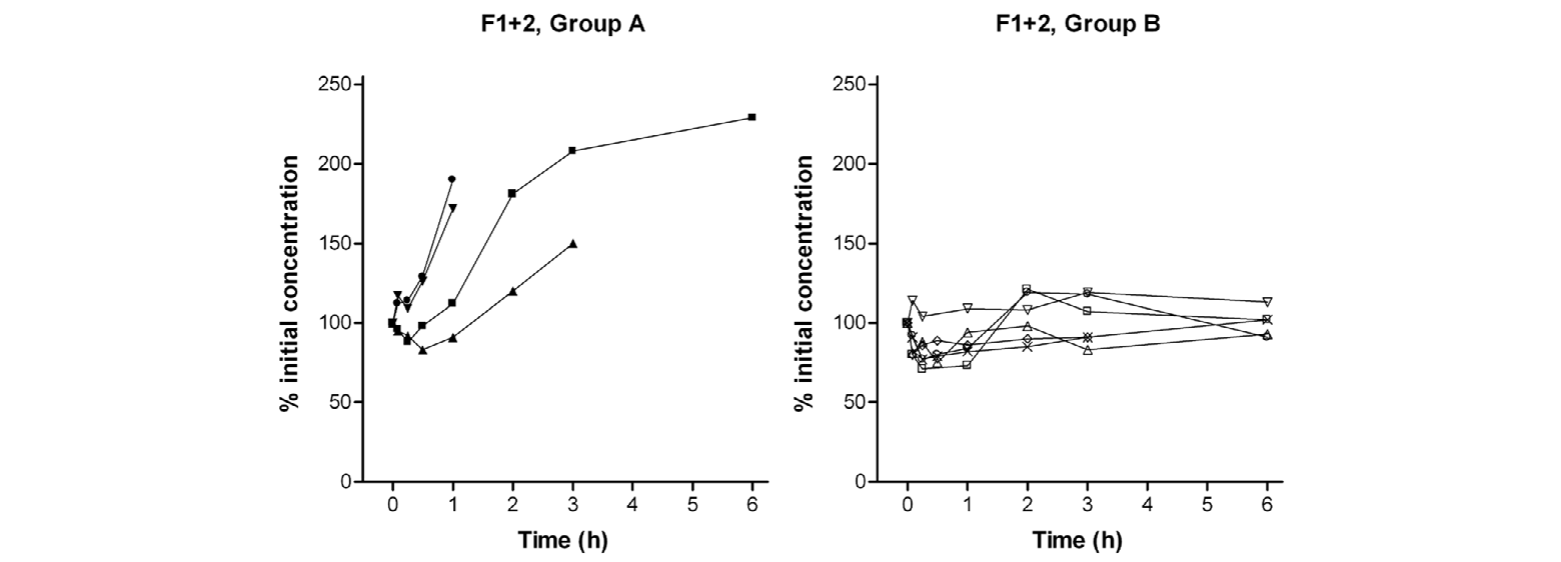
Prothrombin fragment F1+2 during hemofiltration. Curves represent values of individual patients. Group A, patients demonstrating an increase in thrombin generation. Group B, patients with a constant level of thrombin generation. F1+2, prothrombin fragment F1+2.

**Figure 2:**
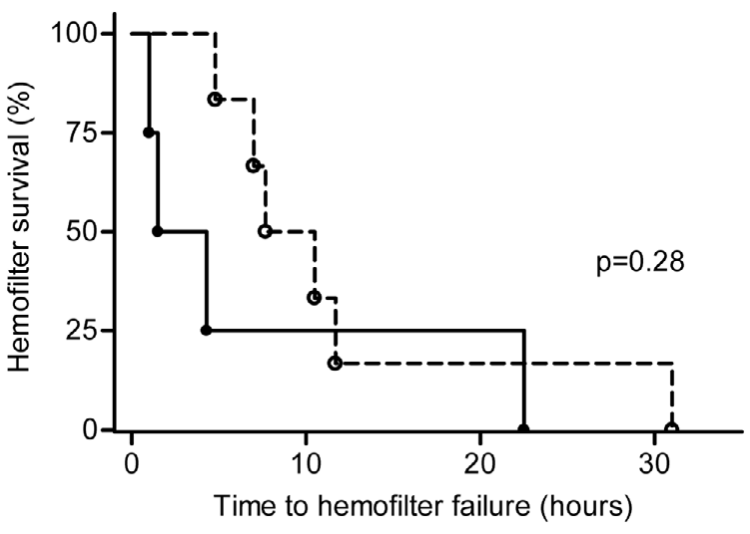
Kaplan–Meier survival function indicating hemofilter survival times. Survival function indicating hemofilter survival times between patients with increased thrombin (group A, closed circles) and patients without increased thrombin generation (group B, open circles).

Nine out of 10 patients showed coagulation activation before the initiation of CVVH, as reflected by increased F1+2 levels. Figure 1 shows the F1+2 levels during CVVH for each patient. The concentrations of F1+2 increased in patients 1, 2, 8 and 9 (defined as group A) and did not change in the other patients (defined as group B) (*P *< 0.001). One hour after the onset of CVVH, the relative increase in the transmembrane pressure was significantly higher (*P *= 0.01) in group A compared with group B (57% (42–80%) in group A and 2% (2–7%) in group B). In group A the lifespan of the circuit was less than 4.3 hours in three patients, but one patient had an unexpected long circuit run of 22.5 hours. The difference in circuit life span was not significantly different between the two groups (Figure 2).

### Baseline coagulation parameters

Coagulation parameters before the initiation of CVVH are presented in Table 2, along with their reference values. By comparison with group B, baseline levels of the activated partial thromboplastin time, the kallikrein–C1-inhibitor complex and the FXIIa–C1-inhibitor complex were significantly lower in group A, whereas the platelet count was significantly higher in group A.

### Coagulation parameters during CVVH

**Figure 3:**
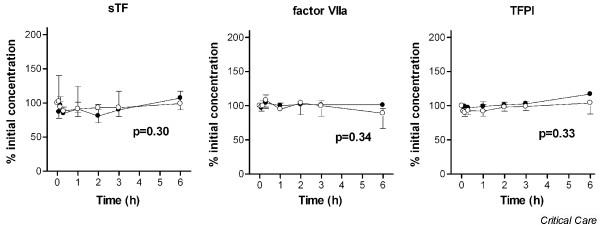
Soluble tissue factor, activated factor VII and tissue factor pathway inhibitor during hemofiltration. Data points are median and interquartile ranges. Closed circles, patients with thrombin generation (group A); open circles, patients without thrombin generation (group B). *P *value represents the difference in time course between both groups by linear mixed models and during the first three hours of hemofiltration. sTF, soluble tissue factor; factor VIIa, activated factor VII; TFPI, tissue factor pathway inhibitor.

**Figure 4:**
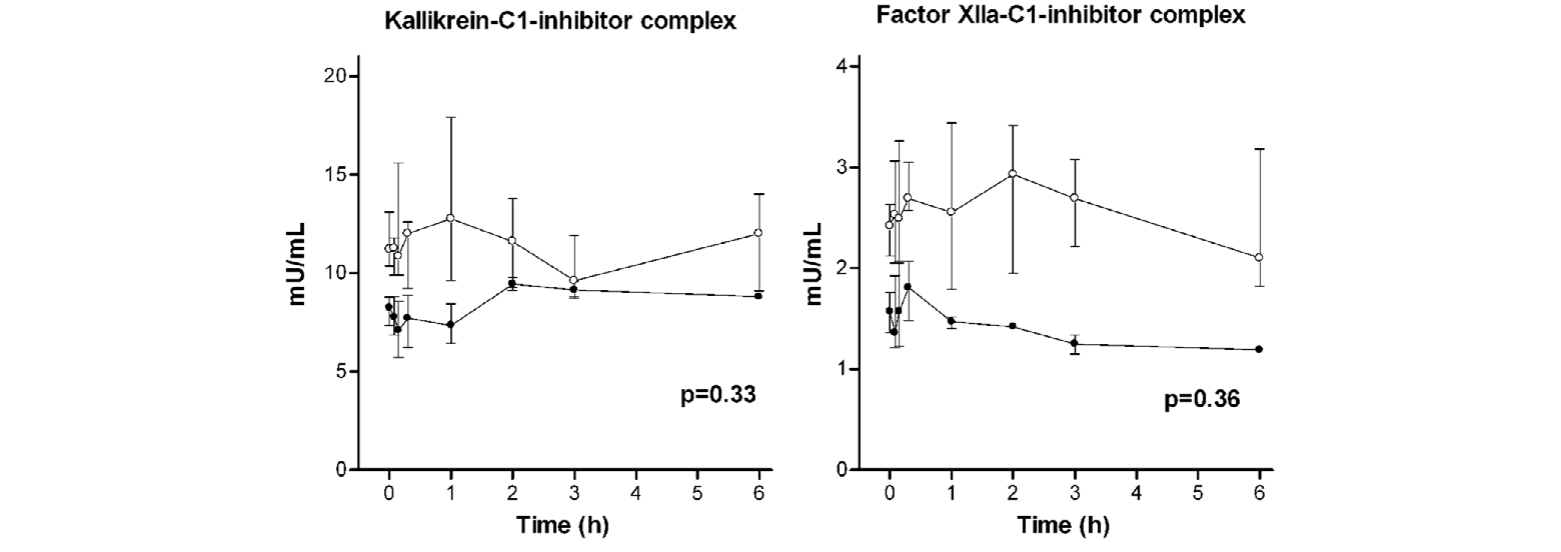
Concentrations of kallikrein–C1 inhibitor and activated factor XII–C1-inhibitor complexes during hemofiltration. Levels are absolute values in order to display the significant (*P *= 0.02) difference at baseline. Data points are median and interquartile ranges. Closed circles, patients with thrombin generation (group A); open circles, patients without thrombin generation (group B). *P *value represents the difference in time course between both groups by linear mixed models and during the first three hours of hemofiltration. Factor XIIa, activated factor XII.

**Figure 5:**
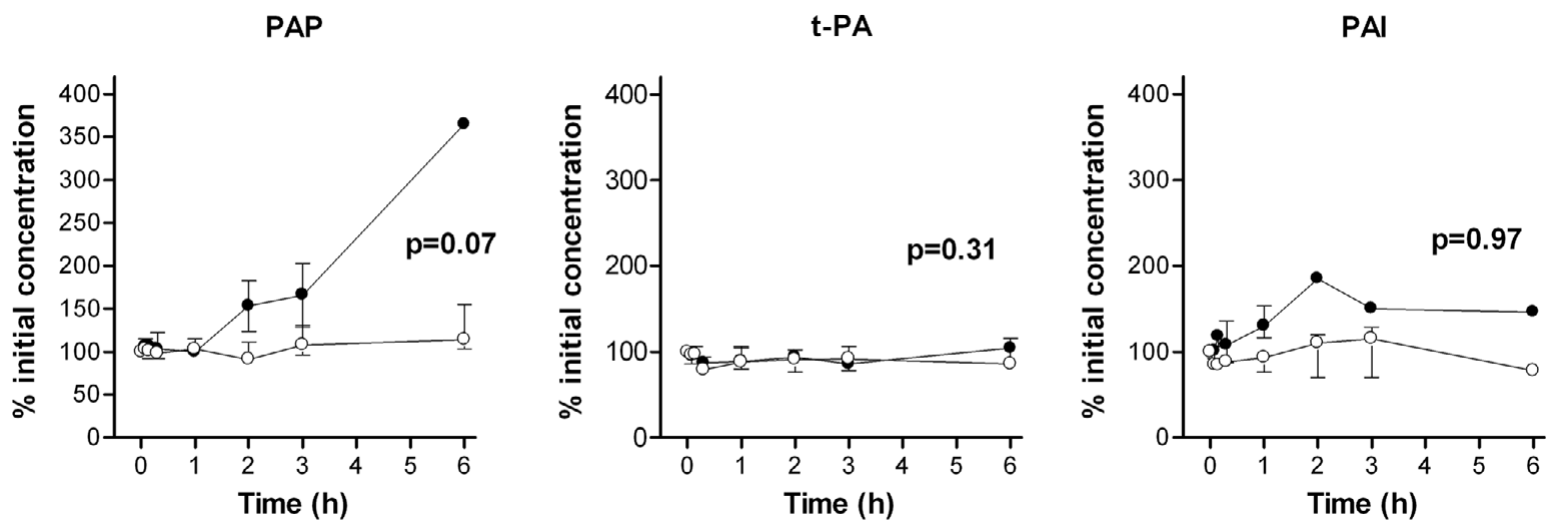
Plasmin–antiplasmin complexes, tissue plasminogen activator and plasminogen activator inhibitor type 1 during hemofiltration. Data points are median and interquartile ranges. Closed circles, patients with thrombin generation (group A); open circles, patients without thrombin generation (group B). *P *value represents the difference in time course between both groups by linear mixed models during the first three hours of hemofiltration. PAP, plasmin–antiplasmin complexes; t-PA, tissue plasminogen activator; PAI, plasminogen activator inhibitor type 1.

The time courses of the coagulation markers are shown in Figures 2, 3, 4. Data points are shown as a percentage of the initial concentration for those markers that were not significantly different at baseline (Figures 3 and 5), whereas data points are shown as absolute values for those markers that were significantly different at baseline (Figure 4). Analysis of the difference in the time course between group A and group B was limited to the first three hours after the start of CVVH, because only one patient in group A was still on CVVH at six hours.

The difference in the time course between groups A and B was not significant for the tissue factor system (Figure 3) and for the contact system (Figure 4). Levels of t-PA and plasminogen activator inhibitor type 1 were also not significantly different between group A and group B during CVVH (Figure 5). The PAP complex levels tended to increase in group A during CVVH (*P *= 0.07).

## Discussion

In the present study in critically ill patients, we investigated the early effects of CVVH without anticoagulation on systemic markers of coagulation activation and fibrinolysis. During the first six hours of CVVH, increased thrombin generation was found in only four out of ten patients. An early increase in transmembrane pressure, indicating filter clotting, was exclusively seen in the four patients with thrombin generation. Premature clotting of the circuit was found in three of these four patients, necessitating replacement of the circuit. CVVH without anticoagulation did not change the systemic concentrations of markers of the intrinsic pathway or the extrinsic pathway, nor did CVVH affect the systemic concentrations of fibrinolysis markers.

Thrombin generation on an artificial surface, such as the filter membrane, has traditionally been attributed to contact activation of the intrinsic pathway of coagulation that starts upon exposure of contact factors (factor XII, high molecular weight kallikrein and prekallikrein) to a negatively charged surface and their subsequent activation. We did not find any change in plasma levels of the FXIIa–C1-inhibitor complex and the kallikrein-C1-inhibitor complex, making initiation of coagulation via this pathway less likely. This finding confirms the results of Salmon and colleagues, who did not find an increase in contact activation during CVVH using a polyacrilonitrile membrane and systemic heparinization [6]. Interestingly, in our study baseline levels of the FXIIa–C1-inhibitor complex and the kallikrein–C1-inhibitor complex were relatively lower in patients with early increased thrombin generation during CVVH. Several authors have described the role of FXIIa and kallikrein in the activation of fibrinolysis [11,12]. Factor XII is able to activate fibrinolysis by three different pathways: it activates prekallikrein, which in turn activates urokinase-type plasminogen activator; following the activation of prekallikrein, the kallikrein generated can liberate t-PA; and factor XII activates plasminogen directly.

The role of contact activation-dependent fibrinolysis *in vivo *is unclear, but a relationship between contact activation-dependent fibrinolysis and thromboembolic complications has been described [13,14]. Low baseline activation of the contact system may therefore be associated with lower fibrinolysis and an increased risk of filter clotting. In our study, however, fibrinolysis during CVVH was not decreased in group A. On the contrary, we observed a trend towards increased PAP levels during CVVH in patients with early clotting of the filter. This PAP level increase is most probably caused by activated coagulation leading to plasmin generation from plasminogen on the formed fibrin. In this respect, therefore, the PAP levels may be more an indication of coagulation than of fibrinolytic activity itself.

Alternatively, one could speculate that patients with higher baseline levels of FXIIa–C1-inhibitor complex and kallikrein–C1-inhibitor complex have higher baseline thrombin generation. Baseline F1+2 levels were higher in group B than in group A, although the difference was not statistically significant, possibly due to the small number of patients. Thrombin is required for activation of the endogenous anticoagulant protein C system [15,16]. In patients with higher levels of FXIIa–C1-inhibitor complex and kallikrein-C1-inhibitor complex, it is conceivable that coagulation activation during CVVH is decreased following increased endogenous anticoagulant activity. Indeed, an anticoagulant effect of thrombin infusion has been reported in a dog model [16]. In the present study, the protein C levels were no different at baseline between the two groups; however, we did not measure the 'activated' protein C levels.

A contribution of the extrinsic pathway to thrombin generation on artificial surfaces is unexpected at first sight since tissue factor is normally not found on the surface of cells in contact with blood. Monocytes, however, can express tissue factor under certain pathophysiologic conditions, mostly associated with increased endotoxin and/or cytokine levels [17]. Based on the measurements of circulating FVIIa, soluble tissue factor and TFPI, we did not find signs of activation of coagulation via the extrinsic pathway. Our findings are in contrast with another study that concluded activation of tissue factor/FVIIa-mediated coagulation took place in critically ill patients treated with CVVH [4]. This conclusion was based on increased levels of thrombin–antithrombin complexes and FVIIa and on decreased levels of TFPI during CVVH. The change in circulating FVIIa and TFPI levels, however, was relative to values just after the start of CVVH with concurrent administration of heparin. No change in FVIIa and TFPI levels was found when they were compared with pre-CVVH values. The observed changes in TFPI and FVIIa in that study may represent the effects of heparin, rather than activation of the tissue factor/FVIIa-mediated pathway of coagulation, because the concentration of TFPI increases after administration of heparin [18], and because high TFPI levels may bind FVIIa. In our study CVVH was performed without administration of heparin, and no changes in markers of tissue factor/FVIIa-mediated coagulation were observed.

What is the mechanism of increased thrombin generation in the absence of detectable activation of the extrinsic coagulation system and intrinsic coagulation system? One explanation could be a lack of sensitivity of the systemic markers such as soluble tissue factor, FVIIa and TFPI. The total volume of blood in the extracorporeal circuit is only approximately 300 ml. The absolute amount of thrombin formation may therefore be too low to lead to detectable increases in plasma levels of precursor proteins, such as soluble tissue factor or FVIIa. In that case, different study designs are needed to show the pathophysiologic mechanism underlying coagulation during CVVH (for example, studies analyzing tissue factor expression on monocytes in prefilter and postfilter samples, or studies directly analyzing the clot formed in the hemofilter).

Alternatively, an increase in systemic coagulation markers could be prevented by the removal of markers across the filter membrane into the ultrafiltrate or secondary to adsorption to the membrane. The high molecular weight (≥ 35 kDa) and polarity of coagulation factors, however, should significantly prevent marker removal during hemofiltration [19]. In our previous *in vitro *hemofiltration study using the same cellulose triacetate membrane as in the present study, we found only minimal filtration of IL-6 (molecular weight, 23–30 kDa) and the calculated sieving coefficient was approximately 0.1 in the predilution mode [20]. In general, the process of adsorption to the membrane is rapidly saturated, but we cannot rule out some adsorption to the membrane during the first hour of CVVH.

Finally, it is also conceivable that alternative pathways of thrombin generation are responsible for filter clotting, including the direct activation of factor X, either on the surface of activated platelets or by the integrin receptor MAC-1 on leukocytes [3].

Low levels of natural anticoagulants have been suggested to contribute to early filter clotting. In the randomized CRRT study by Kutsogiannis and colleagues [21], comparing regional citrate anticoagulation with heparin anticoagulation, decreasing antithrombin levels were an independent predictor of an increased risk of filter failure. In the retrospective study by du Cheyron and colleagues [22] in sepsis patients requiring CRRT and with acquired antithrombin deficiency, anticoagulation with unfractionated heparin plus antithrombin supplementation prevented premature filter clotting. In our own experience, treatment with recombinant human activated protein C obviated additional anticoagulation during CVVH in patients with severe sepsis [23]. In the present study, however, baseline levels of antithrombin and protein C were not extremely low and no significant difference between patients with and without early thrombin generation was found.

Another natural defense mechanism against activated coagulation is the fibrinolytic system, and a disturbance of the normal balance between fibrinolysis and antifibrinolysis might play a role in thrombosis of the CVVH circuit. In our study the difference in PAP complex, t-PA and plasminogen activator inhibitor type 1 levels before and during CVVH were not statistically significant in patients with and without thrombin generation, but our study is limited by the small number of patients.

At baseline, the platelet count was significantly lower and the prothrombin time and activated partial thromboplastin time were significantly longer in those patients without subsequent coagulation activation. The association of a low platelet count with a decreased risk of filter clotting confirms our finding in earlier studies [24]. The association also confirms the findings of Holt and colleagues, who showed an association between the starting activated partial thromboplastin time and the time to circuit clotting [25].

## Conclusion

We conclude that activation of coagulation can be detected in a minority of intensive care patients treated with CVVH without anticoagulation. Systemic concentrations of markers of the tissue factor/FVIIa system and the contact system did not change during CVVH. We suggest that different studies investigating the activation of coagulation directly at the site of the filter are needed to elucidate the mechanism of clot formation during CVVH.

## Key messages

• Early increase in thrombin generation can be detected in a minority of intensive care patients treated with CVVH without anticoagulation.

• In patients without early coagulation activation during CVVH, the baseline levels of the activated partial thromboplastin time, prothrombin time, FXIIa–C1-inhibitor complex and kallikrein–C1-inhibitor complex were more increased, and the platelet count decreased, in comparison with CVVH patients with early coagulation activation.

• Systemic concentrations of markers of the tissue factor/FVIIa system and the contact system did not change during CVVH.

• Early coagulation during CVVH may be related to lower baseline levels of markers of contact activation.

## Abbreviations

CRRT = continuous renal replacement therapy; CVVH = continuous venovenous hemofiltration; ELISA = enzyme-linked immunosorbent assay; F1+2 = prothrombin fragment F1+2; FVIIa = activated factor VII; FXIIa = activated factor XII; IL = interleukin; PAP = plasmin–antiplasmin; TFPI = tissue factor pathway inhibitor; t-PA = tissue type plasminogen activator.

## Competing interests

The authors declare that they have no competing interests.

## Authors' contributions

CSCB, JCMM, ML and EdJ contributed to the conception and design of the study. CSCB and A-CJMdP performed the study. DR, SZ and GW performed the contact system assays, and JCMM and KB performed all the other assays. JCK contributed to the statistical analysis. All authors participated in the study analysis. CSCB drafted the manuscript, with the assistance of AP and EdJ. All authors read and approved the final manuscript.

## Figures and Tables

**Table 1:** Patient characteristics

Patient number	Age (years)	Gender	Diagnosis	APACHE II score^a^	Cause of acute renal failure	Type of acute renal failure	Urea^b ^(mmol/l)	Creatinine^b ^(μmol/l)	Duration of the CVVH circuit studied (hours)	Filter lifespan (hours)	Outcome
Group A											
1	44	Male	Subarachnoidal hemorrhage	29	Nonseptic	Anuric	21.8	432	3	4.3	Died
2	52	Male	Lung cancer and pneumonia	10	Septic	Oliguric	35	148	1	1.0	Died
8	68	Female	Thoracic aortic prosthesis	15	Nonseptic	Nonoliguric	37	415	6	22.5	Survived
9	64	Male	Ruptured abdominal aortic aneurysm	14	Nonseptic	Nonoliguric	46	379	1	1.5	Survived
Group B											
3	65	Male	Ruptured abdominal aortic aneurysm	28	Nonseptic	Anuric	14.2	210	6	10.5	Survived
4	48	Male	Streptococcal sepsis	23	Septic	Oliguric	19.3	367	6	7.7	Survived
5	75	Male	Myocardial infarction	23	Nonseptic	Anuric	11.1	259	6	11.7	Died
6	65	Male	Bowel ischemia	18	Septic	Oliguric	33.8	368	6	7.0	Died
7	67	Male	Non-Hodgkin lymphoma	24	Nonseptic	Nonoliguric	44.8	392	6	31.0	Died
10	75	Male	Peritonitis	23	Septic	Oliguric	23.9	177	3	4.8	Died

Group A, patients with increased thrombin generation; group B, patients without increased thrombin generation. ^a^APACHE II score, acute physiology and chronic health evaluation II score at intensive care unit admission [26]. ^b^Before continuous venovenous hemofiltration (CVVH).

**Table 2:** Baseline levels of coagulation markers

Coagulation marker	Group A	Group B	*P *value	Normal range (reference value)
Prothrombin fragment F1+2 (nmol/l)	2.5 (0.9–3.8)	4.1 (2.3–6.6)	0.20	0.3–1.6
Soluble tissue factor (pg/ml)	126 (73–216)	207 (30–322)	0.59	55–256
Activated factor VII (mU/ml)	61 (14–141)	97 (20–267)	0.52	16–142
Tissue factor pathway inhibitor (ng/ml)	167 (104–192)	127 (56–200)	0.83	39–149
Antithrombin (%)	78 (46–100)	45 (16–81)	0.09	80–140
Protein C (%)	63 (29–164)	41 (16–89)	0.34	65–110
Plasmin–antiplasmin complexes (ng/ml)	682 (635–788)	727 (281–1287)	1.0	221–512
Tissue-type plasminogen activator (ng/ml)	14.3 (7.0–44.6)	11.7 (8.6–51.7)	0.75	1.5–15
Plasminogen activator inhibitor type 1 (ng/ml)	135 (16–275)	526 (129–2191)	0.06	10–70
Kallikrein–C1-inhibitor complex (mU/ml)	8.2 (5.1–9.9)	11.1 (8.5–18.5)	0.02	<0.6
Activated factor XII–C1-inhibitor complex (mU/ml)	1.6 (1.3–1.8)	2.4 (1.8–4.4)	0.02	<0.5
Platelet count (× 10^9^/l)	136 (90–329)	63 (30–101)	0.02	150–350
Prothrombin time (s)	13.5 (12.7–16.9)	17.5 (15.2–26.1)	0.05	10–13
Activated partial thromboplastin time (s)	25 (21–29)	37 (27–57)	0.02	21–27

Group A, patients with increased thrombin generation; group B, patients without increased thrombin generation. Data presented as median (range).
